# Association between ABCB1 C3435T polymorphism and breast cancer risk: a Moroccan case-control study and meta-analysis

**DOI:** 10.1186/s12863-016-0434-x

**Published:** 2016-09-01

**Authors:** Amal Tazzite, Yaya Kassogue, Bréhima Diakité, Hassan Jouhadi, Hind Dehbi, Abdellatif Benider, Sellama Nadifi

**Affiliations:** 1 Genetics and Molecular Pathology Laboratory, Medical school of Casablanca, Casablanca Hassan II University, 19 Rue Tarik Ibnou Ziad, B.P. 9154 Casablanca, Morocco; 2 Mohammed VI Center for Cancer Treatment, Ibn Rochd University Hospital, Casablanca, Morocco

**Keywords:** ABCB1, C3435T, Polymorphism, Breast cancer, Morocco

## Abstract

**Background:**

Breast cancer is the most common cause of cancer death among women. Several studies have investigated the relationship between the C3435T polymorphism of ABCB1 gene and risk of breast cancer; but the results are conflicting. In the present study, we sought to assess the relationship between the C3435T polymorphism in ABCB1 gene and the risk of breast cancer in a sample of the Moroccan population.

**Methods:**

A case control study was performed on 60 breast cancer patients and 68 healthy women. The ABCB1 C3435T polymorphism was analyzed by polymerase chain reaction-restriction fragment length polymorphism (PCR-RFLP) assay. Furthermore, a meta-analysis including 16 studies with 6094 cases of breast cancer and 8646 controls was performed.

**Results:**

Genotype frequencies were 50 % for CC, 33.3 % for CT and 16.7 % for TT in patients and 41.2 % for CC, 48.5 % for CT and 10.3 % for TT respectively in the control group. This difference was not statistically significant. The same trend as observed in the allele distribution between patients and controls (*P* = 0.84). Findings from the meta-analysis showed that the ABCB1 C3435T polymorphism was not associated with an increased risk of breast cancer in the dominant model (OR = 0.907; 95 % CI = 0.767–1.073; *P* = 0.25) as well as in the recessive model (OR = 1.181; 95 % CI = 0.973–1.434; *P* = 0.093) and in the allele contrast model (OR = 1.098; 95 % CI = 0.972–1.240; *P* = 0.133). However, the stratification of studies on ethnic basis showed that the TT genotype was associated with the risk of breast cancer in Asians (OR = 1.405; 95 % CI = 1.145–1.725; *P* = 0.001), Caucasians (OR = 1.093; 95 % CI = 1.001–1.194; *P* = 0.048) and North African (OR = 2.028; 95 % CI = 1.220–3.371; *P* = 0.006).

**Conclusions:**

We have noted that the implication of C3435T variant on the risk of breast cancer was ethnicity-dependent. However, there is no evidence that ABCB1 C3435T polymorphism could play a role in susceptibility to breast cancer in Morocco. Further studies with a larger sample size, extended to other polymorphisms are needed to understand the influence of ABCB1 genetic variants on the risk of breast cancer.

## Background

Breast cancer is the most common cause of cancer death among women and the most frequently diagnosed female cancer [1]. In Morocco, the cancer registries implemented in Rabat and Casablanca have reported a standardized incidence of 39.9 and 49.2 per 100,000 women respectively [2, 3].

The etiology of this disease is not fully understood, although many risk factors have been identified, such as hormonal, environmental and lifestyle factors. In addition, some molecular markers have been found to be associated with the risk of breast cancer. The human multidrug resistance gene *1 (*MDR1/ABCB1*),* localized to chromosome region 7q21, encodes P-glycoprotein (P-gp) a transmembrane transport protein of 170 kDa that acts as an adenosine triphosphate-dependent efflux transporter pump [4]. This protein is expressed in most human tissues such as intestine, liver, bile, kidney, adrenal gland, placenta, brain and breast. It allows the cells to eliminate hydrophobic substrates and anti-cancer drugs [5–7].

To date, thousands of SNPs have been identified in the ABCB1 gene. One of the most important ABCB1 gene polymorphism is 3435C > T (rs1045642) in exon 26, a synonymous polymorphism witch alters gene expression, protein activity and substrate specificity [8–10]. Indeed, subjects with the TT genotype showed a decreased intestinal P-gp expression compared to CC genotype carriers [11].

Several studies have investigated the relationship between the C3435T polymorphism in ABCB1 gene and the risk of breast cancer in different populations, however the results are inconsistent and the relevance of this polymorphism remains confusing [12–26].

To the best of our knowledge, the relationship between the C3435T polymorphism of ABCB1 gene and the risk of breast cancer has not been examined in the Moroccan population. In this manuscript we evaluated the possible influence of ABCB1 C3435T polymorphism on the susceptibility of breast cancer as well as its correlation with the clinical features of Moroccan patients with breast cancer. Secondly, we carried out a meta-analysis on 16 studies involving 6,094 cases of breast cancer and 8,646 controls in order to widely estimate the relationship between this polymorphism and breast cancer risk.

## Methods

### Study population

The present study was performed on 60 unrelated patients with histologically confirmed breast cancer treated between 2009 and 2010 at Mohammed VI Center for Cancer Treatment, Ibn Rochd University Hospital of Casablanca, Morocco. The control group consisted of 68 healthy women without a history of breast or other cancers. The general characteristics of the patients, including age of menarche, age of first pregnancy, number of pregnancies, breastfeeding, oral contraceptives use, the number of abortions, menopause status, smoking status, body mass index (BMI) and family history of breast cancer were collected through structured survey forms. Clinical and pathological features including age at diagnosis, histology type, tumor size, Scarff-Bloom-Richardson (SBR) grade, lymph nodes status and hormone receptor status were obtained from medical records.

The study was approved by the local ethics committee and written informed consent was obtained from each participant.

### Genotyping

Genomic DNA was extracted from peripheral blood using the salting out method [27]. The ABCB1 C3435T polymorphism was analyzed by polymerase chain reaction-restriction fragment length polymorphism (PCR-RFLP) assay using the primer sequences 5′-TTGATGGCAAAGAAATAAAGC-3′ and 5′-CTTACATTAGGCAGTGACTCG-3′. The PCR reaction was performed in a total volume of 25 μl containing 100 ng of genomic DNA, 1× of 5× GoTaq Flexi Buffer (Promega), 1.25 mM MgCl2, 0.2 mM of each dNTP, 0.625 mM of each primer and 0.5U Go Taq DNA polymerase (Promega). PCR program consisted of an initial denaturation at 94 °C for 5 min followed by 35 cycles of 95 °C for 90 s, 55 °C for 60 s, 72 °C for 90 s, and a final extension at 72 °C for 7 min. Controls with known genotypes (homozygous wild-type, homozygous mutant, and heterozygous) were included in each PCR as a reference. PCR products were digested with 10 units of Mbo I restriction enzyme for 16 h. The digested products were separated by 3 % agarose gel electrophoresis after ethidium bromide staining and observed under UV light. The resulting fragments were 130 bp and 76 bp for the Wild-type homozygote CC, 206 bp, 130 bp and 76 bp for the heterozygote CT and 206 bp for Homozygote mutant variant TT.

### Meta-analysis

A literature search of online databases (PubMed, Embase, Scopus, EBSCO…) was conducted until April 1, 2016 using appropriate keywords: “MDR1”, “ABCB1”, “C3435T polymorphism” and “breast cancer”. All languages were searched initially, but only English language studies were selected.

The following criteria were used to select the eligible studies: (a) a case-control study on the association between ABCB1 C3435T polymorphism and breast cancer risk, (2) have an available genotype or allele frequency, and odds ratio (OR) with 95 % confidence interval (CI). Major exclusion criteria were (a) case-only study and review articles and (b) studies without raw data of the C3435T ABCB1 genotypes.

### Statistical analysis

Statistical analysis was performed using SPSS 19.0 software. The chi-squared (*χ*2) test was used to assess the Hardy-Weinberg equilibrium in genotype distribution. OR with 95 % CI was used to assess the strength of the association between ABCB1 C3435T polymorphism and breast cancer risk. Student’s *t*-test and Fisher exact test were used to evaluate the correlation between the studied polymorphism and the clinicopathological parameters. All tests were two-sided and a *p* value less than 0.05 were considered as statistically significant.

The meta-analysis was performed by MedCalc v.11.6.1.0 software. OR with 95 % CI was used to assess the association between the ABCB1 C3435T polymorphism and breast cancer risk. Genetic heterogeneity was tested by Cochran’s (*Q*) test [28]. Random-effects model was used when the *P* value of heterogeneity test is less than 0.05; otherwise, fix-effects model was used.

## Results

The general characteristics of breast cancer patients without any history of smoking are summarized in Table 1. The mean age at diagnosis was 41.5 ± 10.4 years. Mean of BMI was 26.2 kg/m^2^ (range 16.6–43.6 kg/m^2^). The mean age of menarche was 13.5 years old (range 10–18 years) and the mean age of menopause was 48.5 ± 4.9 years old (range 40–59 years). 63.3 % of patients had descendants (2.9 ± 1.6 children), 79.5 % of them breastfeed (22.6 months, range 1–72). Finally, 48.3 % of patients presented a family history of breast cancer. The hormone replacement therapy has not been used by post-menopausal women.

**Table 1:** General characteristics of Moroccan breast cancer patients included in the case–control study

Characteristics	Mean ± SD
Age (years)	41.5 ± 10.4
BMI (kg/m^2^)	26.2 ± 5.5
Age at menarche (years)	13.5 ± 1.7
Age at first birth (years)	23.4 ± 6.1
Number of children	2.9 ± 1.6
Age at menopause (years)	48.5 ± 4.9
	Mean (range)
Median age at diagnosis (years)	38.5 (25–67)
	N (%)
Nulliparous	22 (36.7)

*BMI* body mass index, *SD* standard deviation, *N* number, *%* percentage

Allele and genotype frequencies of ABCB1 C3435T polymorphism in breast cancer patients and controls are summarized in Table 2. In breast cancer patients, the CC genotype was found in 50 %, CT genotype was found in 33.3 % and TT genotype was found in 16.7 %. In the control group, the frequencies of genotypes were 41.2 % for CC, 48.5 % for CT and 10.3 % for TT. This difference was not statistically significant. The genotype distributions among cases and controls were in Hardy-Weinberg equilibrium (*χ*2 = 3.75, *P* = 0.05 for patients and *χ*2 = 0.36, *P* = 0.55 for controls). The allele frequencies in breast cancer patients and healthy controls were 66.7 and 65.4 % for C allele, and 33.3 and 34.6 % for T allele, respectively (OR = 0.95; 95 % CI = 0.56–1.59; *P* = 0.84). The ABCB1 C3435T polymorphism was not significantly associated with increased risk of breast cancer in the additive, dominant and recessive models.

**Table 2:** Genotype distribution and allelic frequencies of ABCB1 C3435T polymorphism among Moroccan breast cancer cases and healthy controls

Variable	Cases (%)	Controls (%)	OR (95 % CI)	*P* value
Genotypes				
CC	30 (50.0)	28 (41.2)	Reference	
CT	20 (33.3)	33 (48.5)	0.57 (0.27–1.21)	0.14
TT	10 (16.7)	7 (10.3)	1.33 (0.44–3.98)	0.61
Dominant model				
CC	30 (50.0)	28 (41.2)	Reference	
CT + TT	30 (50.0)	40 (58.8)	0.7 (0.35–1.41)	0.37
Recessive model				
CC + CT	50 (83.3)	61 (89.7)	Reference	
TT	10 (16.7)	7 (10.3)	1.74 (0.62–4.91)	0.31
Alleles				
C	80 (66.7)	89 (65.4)	Reference	
T	40 (33.3)	47 (34.6)	0.95 (0.56–1.59)	0.84

*%* percentage, *OR* Odd Ratio, *CI* Confidence Interval, *CC* homozygous wild-type, *CT* heterozygous, *TT* homozygous mutant

Table 3 shows the potential association between the C3435T genetic variant and risk factors of breast cancer in patients. There was no evidence of a significant association between this polymorphism and risk factors of breast cancer (*P* > 0.05).

**Table 3:** Association between ABCB1 C3435T genotypes and breast cancer risk factors in Moroccan patients

Variable	Total	C3435T polymorphism genotypes			*P* value
		CC (%)	CT (%)	TT (%)	
BMI	60				
<22 kg/m^2^	17	11 (64.7)	4 (23.5)	2 (11.8)	0.36
≥22 kg/m^2^	43	19 (44.2)	16 (37.2)	8 (18.6)	
Age of menarche (years)	60				
<13	16	9 (56.25)	4 (25.0)	3 (18.75)	0.71
≥13	44	21 (47.7)	16 (36.4)	7 (15.9)	
Age of first pregnancy (years)	40				
<25	23	11 (47.8)	8 (34.8)	4 (17.4)	0.08
≥25	17	10 (58.8)	1 (5.9)	6 (35.3)	
Number of pregnancies	60				
≤1	30	14 (46.7)	12 (40.0)	4 (13.3)	0.51
>1	30	16 (53.3)	8 (26.7)	6 (20.0)	
Number of abortions	60				
≤1	55	28 (50.9)	19 (34.5)	8 (14.5)	0.34
>1	5	2 (40.0)	1 (20.0)	2 (40.0)	
Oral contraceptives use	60				
Yes	35	18 (51.4)	10 (28.6)	7 (20.0)	0.56
No	25	12 (48.0)	10 (40.0)	3 (12.0)	
Family history of breast cancer	60				
Yes	29	14 (48.3)	8 (27.6)	7 (24.1)	0.29
No	31	16 (51.6)	12 (38.7)	3 (9.7)	
Age group (years)	60				
≤40	34	20 (58.8)	10 (29.4)	4 (11.8)	0.25
>40	26	10 (38.5)	10 (38.5)	6 (23.1)	
Menopausal status	60				
Premenopausal	40	22 (55.0)	11 (27.5)	7 (17.5)	0.26
Postmenopausal	20	8 (40.0)	9 (45.0)	3 (15.0)	
Breastfeeding	60				
Yes	31	17 (54.8)	8 (25.8)	6 (19.4)	0.43
No	29	13 (44.8)	12 (41.4)	4 (13.8)	

*BMI* body mass index, *CC* homozygous wild-type, *CT* heterozygous, *TT* homozygous mutant, *%* percentage

Clinical and pathological characteristics of breast cancer patients, according to ABCB1 genotypes are shown in Table 4. Our data suggest that there is no significant association between the ABCB1 C3435T polymorphism and age at diagnosis, menopausal status, histology type, tumor size, SBR grade, lymph node status and hormone receptor status (*P* > 0.05).

**Table 4:** Association between ABCB1 C3435T genotypes and clinico-pathological characteristics of breast cancer in Moroccan patients

Variable	Total	C3435T polymorphism genotypes			*P* value
		CC (%)	CT (%)	TT (%)	
Histology	60				
IDC	54	26 (48.1)	19 (35.2)	9 (16.7)	0.44
ILC	3	1 (33.3)	1 (33.3)	1 (33.3)	
Others	3	3 (100.0)	-	-	
Tumor size	60				
T1	7	2 (28.6)	3 (42.9)	2 (28.6)	0.41
T2	29	15 (51.7)	8 (27.6)	6 (20.7)	
T3	11	8 (72.7)	3 (27.3)	-	
T4	13	5 (38.5)	6 (46.2)	2 (15.4)	
SBR	60				
I	3	2 (66.7)	-	1 (33.3)	0.61
II	45	23 (51.1)	16 (35.6)	6 (13.3)	
III	12	5 (41.7)	4 (33.3)	3 (25.0)	
Node involvement	60				
N-	25	13 (52.0)	7 (28.0)	5 (20.0)	0.78
N+	34	17 (50.0)	12 (35.2)	5 (14.7)	
Progesterone receptors status	60				
PR-	27	16 (59.3)	8 (29.6)	3 (11.1)	0.38
PR+	33	14 (42.4)	12 (36.4)	7 (21.2)	
Estrogen receptors status	60				
ER-	22	14 (63.6)	5 (22.7)	3 (13.6)	0.27
ER+	38	16 (42.1)	15 (39.5)	7 (18.4)	

*CC* homozygous wild-type, *CT* heterozygous, *TT* homozygous mutant, *%* percentage, *IDC* invasive ductal carcinoma, *ILC* invasive lobular carcinoma, *SBR* Scarff–Bloom–Richardson, *ER* estrogen receptor, *PR* progesterone receptor

**Fig. 1:**
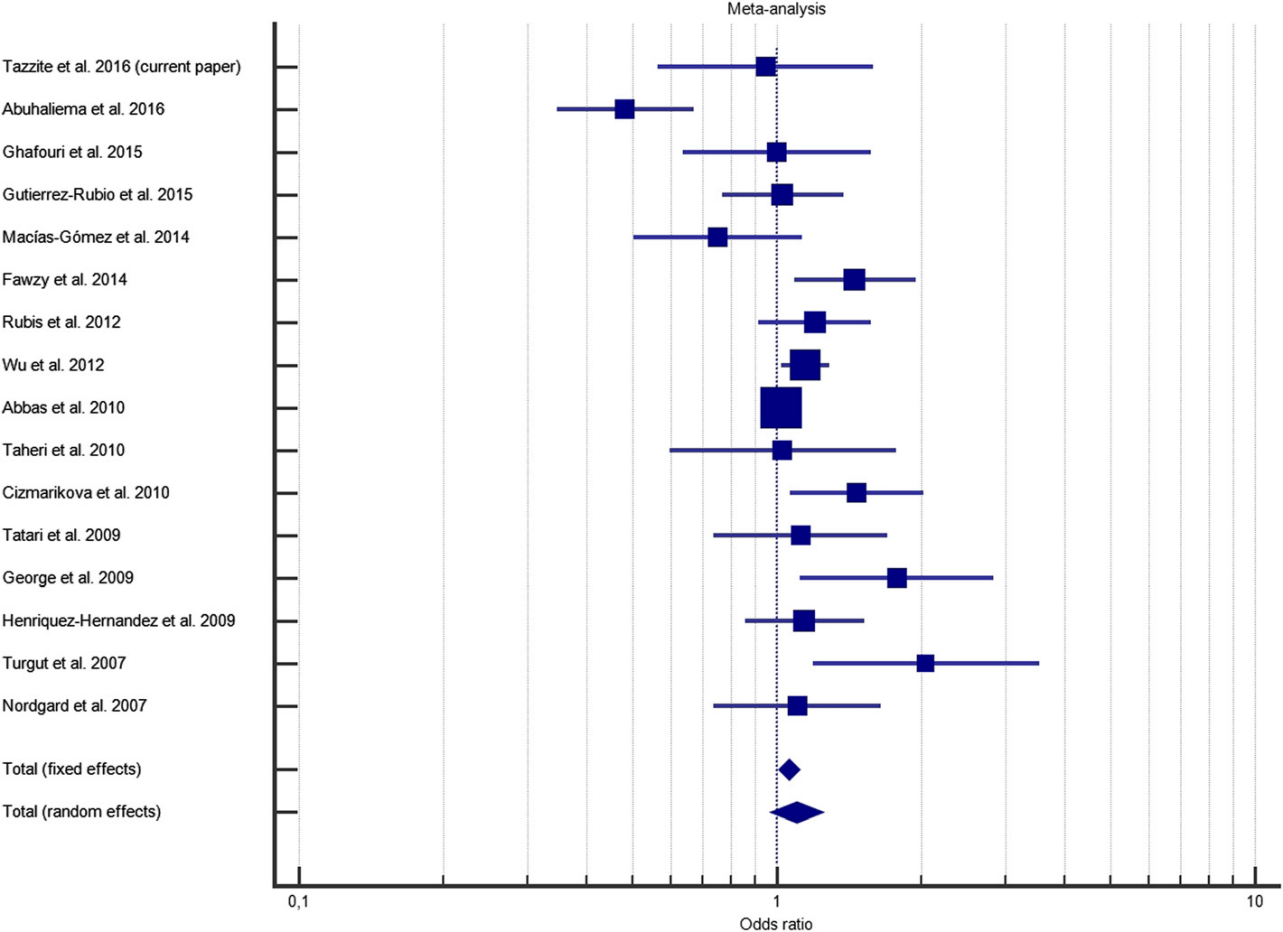
Forest plot of the relationship between ABCB1 C3435T polymorphism and breast cancer risk (T vs. C). The black squares correspond to the odds ratios of the individual studies. The areas of squares are proportional to the study weight. The horizontal lines represent 95 % confidence intervals. The diamonds represent the pooled odd ratios with 95 % confidence intervals

Concerning the meta-analysis, the characteristics of the selected studies are summarized in Table 5. Our findings showed no significant association between C3435T polymorphism of ABCB1 and the risk of breast cancer in the dominant model (OR = 0.907; 95 % CI = 0.767–1.073; *P* = 0.25) as well as the recessive model (OR = 1.181; 95 % CI = 0.973–1.434; *P* = 0.093), and the allele contrast model (OR = 1.098; 95 % CI = 0.972–1.240; *P* = 0.133) (Table 6; Fig. 1).

**Table 5:** Main characteristics of individual studies included in the meta-analysis on ABCB1 C3435T polymorphism and breast cancer risk

Study	Country	Ethnicity	Sample size (N Cases/ N controls)	Genotyping method	Cases					Controls					HWE*
					Genotypes (N)			Alleles (N)		Genotypes (N)			Alleles (N)		
					CC	CT	TT	C	T	CC	CT	TT	C	T	
Tazzite et al. 2016 (current paper)	Morocco	North Africa	60/68	PCR-RFLP	30	20	10	80	40	28	33	7	89	47	0.55
Abuhaliema et al. 2016 [26]	Jordan	Middle East	150/150	PCR-RFLP	68	62	20	198	102	40	65	45	145	155	0.11
Ghafouri et al. 2015	Iran	Caucasian	100/200	PCR-RFLP	75	16	9	166	34	141	50	9	332	68	0.11
Gutierrez-Rubio et al. 2015 [24]	Mexico	Mixed	248/152	PCR-RFLP	82	133	33	297	199	56	72	24	184	120	0.91
Macías-Gómez et al. 2014 [23]	Mexico	Mixed	64/183	PCR-RFLP	15	41	8	71	57	37	103	43	177	189	0.09
Fawzy et al. 2014 [22]	Egypt	North Africa	190/190	ARMS-PCR	60	92	38	212	168	76	94	20	246	134	0.25
Rubis et al. 2012 [21]	Poland	Caucasian	209/205	PCR-RFLP	48	96	65	192	226	52	103	50	207	203	0.94
Wu et al. 2012 [20]	China	Asian	1,173/1,244	PCR-RFLP	388	565	220	1,341	1,005	440	624	180	1,504	984	0.08
Abbas et al. 2010 [19]	Germany	Caucasian	3,148/5,486	MALDI-TOF MS	703	1,543	902	2,949	3,347	1,228	2,736	1,522	5,192	5,780	0.98
Taheri et al. 2010 [18]	Iran	Caucasian	54/50	PCR-RFLP	10	30	14	50	58	10	27	13	47	53	0.55
Cizmarikova et al. 2010 [17]	Slovak	Caucasian	221/113	PCR-RFLP	46	108	67	200	242	35	54	24	124	102	0.71
Tatari et al. 2009 [16]	Iran	Caucasian	106/77	PCR-RFLP	16	57	33	89	123	12	45	20	69	85	0.11
George et al. 2009 [15]	India	Asian	86/68	PCR-RFLP	8	39	39	55	117	15	32	21	62	74	0.67
Henriquez-Hernandez et al. 2009 [14]	Spain	Caucasian	135/301	PCR-RFLP	35	70	30	140	130	85	162	54	332	270	0.13
Turgut et al. 2007 [13]	Turkey	Caucasian	57/50	PCR-RFLP	7	33	17	47	67	18	23	9	59	41	0.73
Nordgard et al. 2007 [12]	Norway	Caucasian	93/109	PCR-RFLP	9	51	33	69	117	17	52	40	86	132	0.99

*N* number, *CC* homozygous wild-type, *CT* heterozygous, *TT* homozygous mutant, *PCR-RFLP* Polymerase chain reaction-restriction fragment length polymorphism, *ARMS-PCR* Amplification refractory mutation system-polymerase chain reaction, *MALDI-TOF MS* Matrix assisted laser desorption/ionization time-of-flight mass spectrometry, *HWE* Hardy Weinberg equilibrium; *P value in the control group

**Table 6:** Pooled analysis for the association between ABCB1 C3435T polymorphism and breast cancer risk

Study	Dominant model CC vs. TT + CT			Recessive model TT vs. CC + CT			Allele contrast model T vs. C		
	OR	95 % CI	*P* value	OR	95 % CI	*P* value	OR	95 % CI	*P* value
Tazzite et al. 2016 (current paper)	1.429	0.710 to 2.875		1.743	0.619 to 4.910		0.947	0.564 to 1.590	
Abuhaliema et al. 2016 [26]	2.280	1.405 to 3.700		0.359	0.200 to 0.645		0.482	0.347 to 0.670	
Ghafouri et al. 2016 [25]	1.255	0.728 to 2.165		2.099	0.806 to 5.466		1.000	0.636 to 1.571	
Gutierrez-Rubio et al. 2015 [24]	0.847	0.555 to 1.292		0.819	0.463 to 1.447		1.027	0.767 to 1.375	
Macías-Gómez et al. 2014 [23]	1.208	0.611 to 2.388		0.465	0.206 to 1.052		0.752	0.502 to 1.127	
Fawzy et al. 2014 [22]	0.692	0.454 to 1.055		2.125	1.185 to 3.811		1.455	1.086 to 1.948	
Rubis et al. 2012 [21]	0.877	0.559 to 1.376		1.399	0.908 to 2.157		1.200	0.914 to 1.577	
Wu et al. 2012 [20]	0.903	0.763 to 1.069		1.365	1.100 to 1.693		1.145	1.021 to 1.285	
Abbas et al. 2010 [19]	0.997	0.897 to 1.108		1.046	0.949 to 1.153		1.020	0.958 to 1.085	
Taheri et al. 2010 [18]	0.909	0.343 to 2.411		0.996	0.414 to 2.395		1.029	0.596 to 1.774	
Cizmarikova et al. 2010 [17]	0.586	0.350 to 0.980		1.613	0.946 to 2.753		1.471	1.066 to 2.030	
Tatari et al. 2009 [16]	0.963	0.427 to 2.173		1.288	0.669 to 2.479		1.122	0.738 to 1.705	
George et al. 2009 [15]	0.362	0.144 to 0.915		1.857	0.953 to 3.618		1.782	1.119 to 2.838	
Henriquez-Hernandez et al. 2009 [14]	0.889	0.562 to 1.408		1.307	0.792 to 2.158		1.142	0.856 to 1.522	
Turgut et al. 2007 [13]	0.249	0.093 to 0.663		1.936	0.773 to 4.848		2.051	1.189 to 3.541	
Nordgard et al. 2007 [12]	0.580	0.245 to 1.371		0.949	0.533 to 1.688		1.105	0.738 to 1.653	
Total (fixed effects)	0.952	0.882 to 1.028	0.209	1.110	1.027 to 1.200	*0.008*	1.059	1.010 to 1.111	*0.017*
Total (random effects)	0.907	0.767 to 1.073	0.250	1.181	0.973 to 1.434	0.093	1.098	0.972 to 1.240	0.133
Test for heterogeneity	Q = 35.1721 DF = 15 I^2^ = 57.35 % *Ph = 0.0023*			Q = 39.4836 DF = 15 I^2^ = 62.01 % *Ph = 0.0005*			Q = 48.4431 DF = 15 I^2^ = 69.04 % *Ph < 0.0001*		

Values in italic are statistically significant (*P* value <0.05); Random effects model was used when Ph < 0.05 otherwise fixed effects model was used

*CC* homozygous wild-type, *CT* heterozygous, *TT* homozygous mutant, *OR* Odd Ratio, *CI* Confidence Interval, *Q* chi-squared statistic, *DF* degrees of freedom; I^2^: percentage of total variation across studies due to heterogeneity; Ph: *P* value of Q test for heterogeneity test

A significant correlation was observed between breast cancer risk and C3435T polymorphism in the recessive model (*P* = 0.008) and in the allele contrast model (*P* = 0.017) under the fixed-effects model. However, we observed heterogeneity among the total studies in the recessive model (I^2^ = 62.01 %; *P* = 0.0005), in the dominant model (I^2^ = 57.35 %; *P* = 0.0023) and in the allele contrast model (I^2^ = 69.04 %; *P* < 0.0001). This explains the use of the random effects model in all these cases.

To identify the potential source of the significant heterogeneity observed in the overall population, we performed a subgroup analysis based on ethnicity. The results showed an increased risk of breast cancer among Asians (OR = 1.405; 95 % CI = 1.145–1.725; *P* = 0.001), Caucasians (OR = 1.093; 95 % CI = 1.001–1.194; *P* = 0.048) and North African (OR = 2.028; 95 % CI = 1.220–3.371; *P* = 0.006) with the TT genotype under the recessive model. Nevertheless, no significant association was found in mixed populations. We noted a significant association with breast cancer risk in the homozygote and allele contrast models for North Africans and Asians populations carrying the TT genotype and T allele (Table 7).

**Table 7:** Stratified analysis based on ethnicity for the association between ABCB1 C3435T polymorphism and breast cancer risk

Ethnicity	Cases/ controls	Recessive model TT vs. CC + CT			Dominant model CC vs. TT + CT			Homozygote model TT vs. CC			Allele contrast model T vs. C		
		OR (95 % CI); *P* value		Ph	OR (95 % CI); *P* value		Ph	OR (95 % CI); *P* value		Ph	OR (95 % CI); *P* value		Ph
		Fixed effects	Random effects		Fixed effects	Random effects		Fixed effects	Random effects		Fixed effects	Random effects	
Asian	4,835/7,334	1.405 (1.145 to 1.725) ; *0.001*	1.405 (1.144 to 1.725) ; *0.001*	0.3888	0.876 (0.742 to 1.033) ; 0.116	0.644 (0.271 to 1.528) ; 0.318	0.0573	1.458 1.156 to 1.840) ; *0.001*	1.918 (0.809 to 4.545) ; 0.139	0.0816	1.175 (1.051 to 1.313) ; *0.005*	1.346 (0.887 to 2.042) ; 0.163	0.0706
Caucasian	4,123/6,591	1.093 (1.001 to 1.194) ; *0.048*	1.124 (1.002 to 1.262) ; *0.047*	0.3993	0.954 (0.869 to 1.049) ; 0.333	0.844 (0.677 to 1.052) ; 0.131	0.0857	1.119 (1.000 to 1.252) ; 0.050	1.427 (1.079 to 1.886) ; *0.013*	0.0836	1.054 (0.996 to 1.114) ; 0.068	1.147 (1.014 to 1.298) ; *0.030*	0.1382
Mixed	312/335	0.666 (0.422 to 1.054) ; 0.083	0.667 (0.391 to 1.138) ; 0.137	0.2640	0.933 (0.651 to 1.339) ; 0.708	0.935 (0.653 to 1.338) ; 0.712	0.3854	0.755 (0.449 to 1.269) ; 0.289	0.724 (0.368 to 1.422) ; 0.348	0.2221	0.923 (0.729 to 1.169) ; 0.506	0.908 (0.673 to 1.225) ; 0.528	0.2197
North Africa	250/258	2.028 (1.220 to 3.371) ; *0.006*	2.026 (1.218 to 3.369) ; *0.007*	0.7438	0.841 (0.587 to 1.204) ; 0.343	0.940 (0.466 to 1.896) ; 0.864	0.0822	2.072 (1.196 to 3.590) ; *0.009*	2.072 (1.193 to 3.596) ; *0.010*	0.3610	1.312 (1.018 to 1.690) ; *0.036*	1.241 (0.826 to 1.863) ; 0.298	0.1572

Values in italic are statistically significant (*P* value <0.05); Random effects model was used when Ph < 0.05 otherwise fixed effects model was used

*CC* homozygous wild-type, *CT* heterozygous, *TT* homozygous mutant, *OR* Odd Ratio, *CI* Confidence Interval; Ph: *P* value of Q test for heterogeneity test

## Discussion

ABCB1 gene is a member of the ABC family that encodes P-gp protein, which is an ATP-dependent efflux pump that allows the cells to eliminate toxins and carcinogenic substances [6]. Some reports suggested that this polymorphism may influence the risk of a number of cancers, especially breast carcinoma [29]. Indeed, this synonymous mutation (Ile1145Ile) influences protein stability [30] and causes cellular damage or apoptosis alteration witch play an important role in cancer development due to an accumulation of metabolites within the cell [31, 32].

In the present study, we have evaluated the association between the genetic polymorphism C3435T of ABCB1 gene and the risk of breast cancer among Moroccan patients. In agreement with a number of previous reports [16, 18, 21, 23, 24], our findings revealed no significant association between this polymorphism and breast cancer development. Otherwise, Gutierrez-Rubio et al. [24] did not find differences in the distribution of C3435T polymorphism between breast cancer patients and controls. However, when they have examined the association between this polymorphism and breast cancer risk, according to the menopausal status of patients, they found that premenopausal women with T allele have 2-fold increased risk of breast cancer.

In contrast, other studies have reported different results. Most of these findings reported the association of TT genotype and T allele with high risk of breast cancer. Turgut et al. [13] revealed a 1.5-fold increased risk for the development of breast cancer in T allele carriers. Similarly, Cizmarikova et al. [17] and George et al. [15] have found a significantly higher prevalence of T allele and TT genotype in breast cancer patients when compared to controls (*P* = 0.019 and *P* = 0.025 respectively). Furthermore, Wu et al. [20] conducted a large study with 1,173 breast cancer women and 1,244 controls and reported a significant increase in the frequency of the TT genotype (TT vs. CC: OR = 1.386; 95 % CI = 1.091–1.761; *P* = 0.008) and T allele (OR = 1.281; 95 % CI = 1.021–1.285; *P* = 0.020) in patients with breast cancer. More recently, Fawzy et al. [22] studied 190 Egyptian females with breast cancer and showed that the frequency of the TT genotype (OR = 1.45; 95 % CI = 1.09–1.94; *P* = 0.01) and T allele (OR = 2.41; 95 % CI = 1.27–4.56; *P* = 0.0006) were significantly higher in breast cancer patients compared to healthy controls.

Counterwise, a recent study of Abouhalima et al. [26] among Jordanian women have revealed a higher prevalence of CC genotype in breast cancer patients compared to controls (*P* < 0.001) and individuals with T allele were 2 times less likely to develop breast cancer (*P* < 0.0001). It should be noted that in a study of Salem et al., the T allele was more prevalent among Jordanians than the C allele [33]. On the other hand, the authors suggested strong linkage disequilibrium with other polymorphisms in ABCB1 gene and alterations in the post translational pathway which influences the efficacy and stability of P-gp in patients with CC genotype [26]. Similarly, in Kurdish patients the frequency of CC genotype and C allele were higher in patients than in controls; this result was not statistically significant [25].

These conflicting results may be due to the ethnicity and the environment of the studied population, the analysis type and the sample size. Indeed, it was reported that the distribution of C3435T genotypes varies among populations [33–35]. In Caucasians, the C3435T genotype frequency was 22, 50 and 28 % for CC, CT and TT genotypes respectively [36]. In Morocco, genotype frequencies were 39 % for CC, 51 % for CT and 10 % for TT [37].

Besides, we did not find a significant difference in the distribution of breast cancer risk factors among CC, CT and TT genotypes. Similar to our results, Tatari et al. reported an absence of association between C3435T genotypes of ABCB1 gene and the risk factors, including age of disease onset, cancer stage, family history of the cancer, smoking history, age of menarche, age of first pregnancy, number of pregnancies, abortion history, and history of oral contraceptive consumption (*P* > 0.05) [16]. Also, Wu et al. have reported no statistically significant correlation between genotype distributions and age at diagnosis, menopausal state and family history of breast carcinoma [20].

The correlation between the clinical and pathological features of breast cancer in the present study, according to C3435T polymorphism genotypes revealed no significant association at this level. In this line, Turgut et al. [13], Wu et al. [20] and Macías-Gómezdid et al. [23] have reported similar results. However, Ghafouri et al. have found a significant correlation between ABCB1 C3435T polymorphism and clinical grades of breast cancer with higher grade in CC carriers (*P* = 0.027) [25]. On the other hand, Wu et al. [20] observed that patients with a negative status of ER and PR have more CT + TT genotypes than CC genotype (*P* = 0.013).

In second place, we tried through the present study to evaluate the association between the ABCB1 C3435T polymorphism and breast cancer risk through a meta-analysis involving 16 studies with 6,094 cases of breast cancer and 8,646 controls. Our meta-analysis suggests that the ABCB1 C3435T polymorphism has no effect on breast cancer development. It is noteworthy that a lack of homogeneity between studies was observed regarding the distribution of ABCB1 C3435T polymorphism. This heterogeneity might be explained by ethnicity variability and sample size across the different studies included in the present meta-analysis. Indeed, Wang et al. in a meta-analysis observed significant heterogeneity among the total studies, but not in the small size sample analysis [38].

A number of meta-analysis were undertaken to assess the association between ABCB1 C3435T polymorphism and risk of breast cancer [38–41]. The first meta-analysis conducted in 2011 which included 7 studies for ABCB1 C3435T polymorphism did not show any association between this polymorphism and risk of breast cancer [38]. However, it should be noted some errors in C3435T genotypes reported for the study of Nordgard et al. [12] and George et al. [15] which probably would have influenced the study results [42].

A meta-analysis conducted two years later [38], enrolled 10 case-control studies with 5,282 cases and 7,703 controls, indicated that this polymorphism were associated with a significantly increased risk of breast cancer according to the following models TT vs. CC (*P* = 0.003); TT vs. CT + CC (*P* = 0.003) and TT + CT vs. CC (*P* = 0.029). Although our study was based on the same data of the previous meta-analysis [38], we did not find any significant association between the C3435T polymorphism and risk of breast cancer. This can be explained by the fact that we have added the results from other populations with different genetic background, such as North Africa (Morocco, Egypt), Middle East (Jordan) and also mixed populations (Mexico). In Morocco, for example, the frequency of the wild-type 3435CC genotype was found to be higher than that observed in Caucasians and Asians. Conversely, the frequency of the mutated homozygous variant was lower compared to the same populations. However, similar results were reported in Egypt which may be attributed probably to their common ethnic and geographic origins [37].

Thereby, we stratified our meta-analysis by ethnicity to get a better idea about the involvement of this polymorphism in breast cancer risk. Our findings indicate that patients with TT genotype had a significantly increased risk of breast cancer in Asians, Caucasians and North African but not among mixed populations. These might be due to the differences in genetic background and lifestyle and seem to confirm that the C3435T polymorphism of ABCB1 gene varies across different populations [43].

## Conclusions

To the best of our knowledge, this is the first study, which examined the association of ABCB1 C3435T polymorphism with the risk of breast cancer in a sample of the Moroccan population. The results of the present study revealed no difference between breast cancer patients and controls for ABCB1 C3435T polymorphism. In addition, we did not find a significant correlation between this polymorphism and clinicopathological features of breast cancer patients. This may be explained by the limited statistical power due to our small sample size. It is also necessary to remember that there are other polymorphisms in the ABCB1 gene implicated in the etiology of breast cancer which also deserve to be studied. Therefore, the results of the present study must be interpreted with caution and cannot be generalized. Larger case-control study, with at least 340 breast cancer patients and 340 healthy controls, including more polymorphisms of ABCB1 with haplotype analysis is needed to approve or not our conclusions and to obtain more clear information about the influence of ABCB1 genetic variants in breast cancer risk in Morocco. Moreover, it would also be interesting to study the association of this polymorphism with chemotherapy resistance in breast cancer in our population. Furthermore, the results obtained from the meta-analysis demonstrated that the implication of C3435T variant on the risk of breast cancer risk was modulated by ethnicity.

## Abbreviations

ABCB1: ATP-binding cassette sub-family B member 1
BMI: Body mass index
CI: Confidence interval
ER: Estrogen receptor
OR: Odds ratio
PCR-RFLP: Polymerase chain reaction-restriction fragment length polymorphism
PR: Progesterone receptor
SBR: Scarff-Bloom-Richardson
